# Screening and managing cannabis use: comparing GP’s and nurses’ knowledge, beliefs, and behavior

**DOI:** 10.1186/1747-597X-7-31

**Published:** 2012-07-24

**Authors:** Melissa M Norberg, Peter Gates, Paul Dillon, David J Kavanagh, Ramesh Manocha, Jan Copeland

**Affiliations:** 1 National Cannabis Prevention and Information Centre, UNSW, PO Box 684, Randwick, NSW, 2031, Australia; 2 School of Psychology and Counselling, Institute of Health and Biomedical Innovation, Queensland University of Technology, Kelvin Grove, QLD, 4059, Australia; 3 Department of Psychiatry, Sydney Medical School, Level 5 Building 36, Royal North Shore Hospital, St Leonards, NSW, 2065, Australia

**Keywords:** Cannabis, Marijuana abuse, Diagnosis, Therapeutics, Primary health care, Attitude

## Abstract

**Background:**

General practitioners (GPs) and nurses are ideally placed to address the significant unmet demand for the treatment of cannabis-related problems given the numbers of people who regularly seek their care. The aim of this study was to evaluate differences between GPs and nurses’ perceived knowledge, beliefs, and behaviors toward cannabis use and its screening and management.

**Methods:**

This study involved 161 nurses and 503 GPs who completed a survey distributed via conference satchels to delegates of *Healthed* seminars focused on topics relevant to women and children’s health. Differences between GPs and nurses were analyzed using χ^2^- tests and two-sample *t*-tests, while logistic regression examined predictors of service provision.

**Results:**

GPs were more likely than nurses to have engaged in cannabis-related service provision, but also more frequently reported barriers related to time, interest, and having more important issues to address. Nurses reported less knowledge, skills, and role legitimacy. Perceived screening skills predicted screening and referral to alcohol and other drug (AOD) services, while knowing a regular user increased the likelihood of referrals only.

**Conclusions:**

Approaches to increase cannabis-related screening and intervention may be improved by involving nurses, and by leveraging the relationship between nurses and doctors, in primary care.

## Background

Cannabis remains the most commonly used illicit drug, with around 200 million current users worldwide [1]. Cannabis use increases the risk of chronic respiratory and cardiovascular problems [2-5], and around one in nine users are at risk of developing dependence [6,7]. In Australia, these health risks are of significant concern given that cannabis use contributes to 10% of the burden relating to illicit drug use [8]. While few cannabis users seek specialist drug treatment [9,10], general practitioners (GPs) are the most frequently sought resource for treatment of cannabis use [11]. As approximately 80% of Australians visit a GP at least once a year [12], primary care provides substantial opportunity for cannabis use screening and intervention. Doctors and nurses in primary care may be able to influence their patients’ cannabis use through a variety of health strategies [13], but their attitudes, knowledge, and skills may limit the provision of such care [14,15].

Although primary care practitioners are encouraged to, and typically believe it is appropriate for them to screen and provide early interventions for substance use [16], many do not feel comfortable diagnosing or treating substance use problems [17-19]. Further, the content of a typical substance use brief intervention is not well known [16] and commonly disregarded as ineffective [18,20]. Many doctors and nurses have reported avoiding substance use discussions due to anticipated negative reactions from patients, believing that patients will not be honest about their substance use, not having enough training and resources, and time constraints [18,20-22]. Conversely, doctors and nurses report that having readily available support services, quick and easy screening instruments and counseling material, more training, and evidence of the successful impact for early intervention would lead to their greater involvement in screening and intervention [16,20,22].

If more cannabis screening and intervention were to occur within primary care, a key question is whether nurses or GPs could more feasibly undertake this work. While nurses may be less subject to the time constraints for opportunistic intervention that doctors experience [23,24], findings from the alcohol literature indicate that nurses compared to GPs have less knowledge and skills about screening and intervention [16,25], have less favorable attitudes towards discussing substance use with their patients [16], and are less likely to believe that this work should be a part of their role [16]. In addition, qualitative research found that nurses can be hesitant to provide alcohol interventions because of their own use and enjoyment of alcohol and because of their beliefs that alcohol use may serve beneficial social and coping functions for some patients [21]. If these alcohol-related findings are transferrable to cannabis use, they suggest that nurses may be less inclined than GPs to screen for cannabis use and provide early intervention.

While a modest amount of research has examined GP’s and nurses’ attitudes towards alcohol and substance use in general, relatively little has focused specifically on cannabis use. In a UK survey of 97 GPs, most believed that cannabis use posed a health risk and may lead to mental health problems, but less than half believed that they had adequate knowledge about cannabis use, and only a third felt confident in their ability to advise patients about their cannabis use [22]. In addition, around half believed that GPs should refer patients with cannabis-related problems to specialist drug services, with only a third believing it was appropriate to treat cannabis dependence in primary care. GPs who believed it was appropriate to treat cannabis dependence in primary care felt more confident in their ability to advise cannabis-using patients, whereas GPs who believed cannabis-using patients should be referred to specialist drug services reported a greater need to improve their knowledge of cannabis-related risks.

The aim of this study was to assess potential differences between GPs and nurses. Based on findings reviewed above, we hypothesized that while few GPs and nurses would be confident in their knowledge and skills about cannabis use and its treatment, nurses would report having less training (Hypothesis 1) and less role legitimacy for screening and treating cannabis use (Hypothesis 2), and perceive their knowledge and skills to be poorer than GPs (Hypothesis 3). In addition, we hypothesized that GP’s and nurses’ training, personal experience, knowledge, attitudes, and beliefs would be related to whether or not they engaged in screening or intervention for cannabis use (Hypothesis 4).

## Methods

### Participants

The study sample was comprised of delegates attending a *Healthed* seminar in one of five Australian cities (Sydney, Melbourne, Adelaide, Brisbane, and Perth) in 2011. Each *Healthed* seminar featured approximately sixteen lectures on women and children’s health and represented a rare opportunity to meet with several hundred GPs and nurses in one location. GPs and nurses received professional education points for attending the lectures. Of the 1,925 delegates who attended the *Healthed* seminars, 664 (35%) completed the study survey; 503 surveys were completed by GPs (76%) and 162 were completed by nurses (24%). This distribution was representative of the *Healthed* population, in which 71% were GPs and 26% were nurses. The study sample was predominately female (*n* = 527, 79%), which again reflects the *Healthed* population, as only 41% of Australian GPs are female [26]. The sample had a mean age of 48 years (*SD* = 9.88, range 21-81) and most practiced in a metropolitan area (*n* = 463, 71%). GPs were significantly more likely to be male (27% vs. <1%; χ^2^(1) = 51.23, *p* < 0.001), and younger (GPs *M*_*age*_ = 47.61 years, *SD* = 10.25; nurse’s *M*_*age*_ = 49.93 years, *SD* = 8.04; *t*(343.77) = -2.95, *p* = 0.003), and reported spending significantly more hours in the clinic per week than did nurses (GP *M*_*hours*_ = 30.45, *SD* = 12.65; nurse’s *M*_*hours*_ = 26.56, *SD* = 12.04; *t*(646) = 3.35, *p* = 0.001.

### Data collection

During the opening of each *Healthed* seminar, we informed delegates that the survey and its information and consent form were located in their conference satchel. We advised delegates that if they were interested in participating that they should complete the entire self-report survey, as item nonresponse would render their survey invalid given the brevity of the survey. We instructed delegates to turn in their surveys, before afternoon tea, into dropboxes at the conference. Lastly, we instructed delegates that participation would result in entry into a lottery held during afternoon tea to win one of five gift certificates ($75 AUD). These procedures reflect a cross-sectional design.

### Survey

The survey was devised primarily for use in this study, and thus, had not been psychometrically validated. The survey had 31 items; of which 25 were relevant to the current study. Five items addressed basic demographic information (career, gender, age, physical location of practice, and weekly clinical hours), and two items covered exposure to cannabis issues (relevant training and personal experience with a regular cannabis user). Five items assessed knowledge regarding cannabis use (e.g., *Cannabis users are more likely to have a mental health problem than those who do not use cannabis)*; four of these items were rated on a Likert scale from 0 (*disagree completely)* to 4 (*agree completely)*. The fifth knowledge item was answered with a percentage (*What percentage of people who try cannabis will someday develop cannabis dependence?*).

The remaining 13 items used in this study were based on previous surveys assessing health practitioners’ beliefs, attitudes, and behavior regarding substance use and its treatment. Three items required GPs and nurses to assess their own knowledge about cannabis use and their skills related to screening and managing cannabis use [16-18,27]. These three items were rated on a Likert scale from 0 (*very poor)* to 4 (*very strong)*. In addition, three items assessed beliefs regarding role legitimacy—factors related to the appropriateness of a GP or nurse to intervene with someone’s cannabis use [21,25,27,28] and were rated on a Likert scale from 0 (*disagree completely)* to 4 (*agree completely)*. One item assessed attitudes regarding cannabis use policy (*Cannabis use should be illegal / decriminalized / available for medical use*) [22,29]. Four open-ended items assessed behavior related to screening, intervention, and referral [22,28,30]. Participants responded to these items with numbers (e.g., *I have screened ____ patients for cannabis use in the last month*). The final two items assessed barriers and facilitators to screening and treatment provision [18,20,25,27-29]. These items were answered by checking all applicable barriers or facilitators. Options referred to attitudes, motivation, confidence, and support.

### Statistical analyses

Differences between GPs and nurses on categorical variables were analyzed using 2 Χ 2 χ^2^- tests (e.g., Hypothesis 1), while two-sample *t*-tests were used for continuous variables (e.g., Hypothesis 2, Hypothesis 3). When Levene’s test for equality of variances indicated that the variances between GPs and nurses responses were significantly different, a two-sample *t*-test was performed that did not assume equal variances. Logistic regressions using a generalized linear function examined the prediction of service provision (Hypothesis 4; 0 = none, 1 = at least once; reference category = 0). Before conducting the logistic regressions, zero-order correlations were conducted to determine which variables were appropriate for model inclusion. Only items statistically significant at the univariate level were included at the multivariate level. The significance level for all statistical tests was set at *p* < 0.01 to reduce Type I errors associated with multiple testing.

## Results

### Overview of the study sample

Table 1 contains summary data for cannabis-related training and exposure to cannabis users. GPs were significantly more likely to not have any personal experience with cannabis use compared to nurses (*p* < 0.001), while nurses were more likely to have a relative (*p* < 0.001) or know a friend (*p* < 0.001) who regularly uses cannabis. Further, GPs were significantly more likely to have received a small amount of training in cannabis-related issues (*p* = 0.006), while nurses were more likely to have not received any cannabis-related training (*p* < 0.001).

**Table 1:** Exposure to Cannabis Use

**Item**	***n***	**GPs**	**Nurses**	**χ**^**2**^**(1)**
		***n*(%)**	***n*(%)**	
Cannabis Related Training	660			
None		132 (27%)	66 (41%)	12.26**
Small Amount		300 (60%)	77 (48%)	7.51*
Moderate Amount		60 (12%)	17 (11%)	0.25
Substantial Amount		7 (1%)	1 (<1%)	0.62
Great Deal		0 (0%)	0 (0%)	--
Cannabis users known to participants^a^	657			
No one		331 (67%)	68 (43%)	28.48**
Relative (other than child)		43 (9%)	35 (22%)	20.23**
Child		13 (3%)	7 (4%)	1.27
Friend		129 (26%)	65 (41%)	12.52**
Self		1 (<1%)	0 (0%)	0.32

*Note.*^a^Percentages do not add up to 100% as some participants choose more than one response option.

*p < 0.01, **p ≤ 0.001.

### Knowledge about Cannabis use and its treatment

As set out in Table 2, GPs were more likely than nurses to have agreed that effective psychological treatments exist for reducing cannabis use (*p* = 0.001) and that cannabis users are more likely to have a mental health problem than non-cannabis users (*p* < 0.001). Further, 34% (*n* = 221) of health practitioners somewhat or completely agreed that effective pharmacological treatments exist for cannabis use.

**Table 2:** Knowledge and Beliefs about Cannabis Use and its Treatment

**Item**	**Disagree completely**	**Somewhat disagree**	**Neutral**	**Somewhat agree**	**Agree completely**	***t***	***df***
	***n *(%)**	***n *(%)**	***n *(%)**	***n *(%)**	***n *(%)**		
*Knowledge*							
Cannabis users are more likely to have a mental health problem than those who do not use cannabis							
GPs (*N* = 502)	4 (<1%)	11 (2%)	19 (4%)	159 (32%)	309 (62%)		
Nurses (*N* = 160)	4 (3%)	6 (4%)	20 (13%)	64 (40%)	66 (41%)	4.54**	224.80
Effective psychological treatments exist for helping people to reduce their cannabis use							
GPs (*N* = 501)	9 (2%)	31 (6%)	118 (23%)	196 (39%)	147 (29%)		
Nurses (*N* = 159)	7 (4%)	14 (9%)	42 (26%)	73 (46%)	23 (14%)	3.49**	658
Effective pharmacological treatments exist for assisting with cannabis withdrawal							
GPs (*N* = 499)	43 (9%)	93 (19%)	195 (39%)	116 (23%)	52 (10%)		
Nurses (*N* = 159)	11 (7%)	20 (13%)	75 (47%)	41 (26%)	12 (8%)	-0.65	656
Withdrawal can be a barrier to quitting cannabis							
GPs (*N* = 500)	10 (2%)	23 (5%)	67 (13%)	220 (44%)	180 (36%)		
Nurses (*N* = 158)	2 (<1%)	3 (2%)	32 (20%)	71 (45%)	50 (32%)	0.44	656
*Role Legitimacy*							
Conducting a 10 minute brief assessment of someone’s cannabis use can lead to reductions in their use							
GPs (*N* = 501)	22 (4%)	24 (5%)	105 (21%)	210 (42%)	140 (28%)		
Nurses (*N* = 159)	31 (20%)	26 (!6%)	37 (23%)	47 (30%)	18 (11%)	7.72**	223.44
People in my position are effective in treating patients with cannabis use problems							
GPs (*N* = 499)	22 (4%)	60 (12%)	103 (21%)	218 (44%)	96 (19%)		
Nurses (*N* = 160)	23 (14%)	36 (23%)	52 (33%)	38 (24%)	11 (7%)	7.64**	657
People in my position should receive education about cannabis							
GPs (*N* = 501)	1 (<1%)	1 (<1%)	14 (3%)	116 (23%)	369 (74%)		
Nurses (*N* = 160)	0 (0%)	1 (<1%)	6 (4%)	43 (27%)	110 (69%)	1.20	659

*Note*. *p < 0.01, **p ≤ 0.001.

On average, GPs believed that 21% (*SD* = 21.58) of people who try cannabis will someday develop cannabis dependence, which was significantly less than nurses believed (*M* = 25%, *SD* = 21.58), *t*(131.51) = -1.14, *p* = 0.001. Of the 516 participants who responded to this item, only 24% (*n* = 126) responded with an estimate close to 9% (6-12%) [6,7]. Twenty-one percent of participants did not answer the question.

### Role legitimacy

Nurses reported significantly less role legitimacy about providing cannabis-related services. Compared to nurses, GPs more strongly agreed that conducting a 10-minute brief assessment of someone’s cannabis use can lead to reductions in cannabis use (*p* < 0.001) and that people in their position are effective in treating patients with cannabis use problems (*p* < 0.001). Almost all GPs and nurses agreed that people in their position should receive education regarding cannabis (See Table 2).

### Self-assessment of knowledge and skills

Few GPs and nurses felt confident in their knowledge and skills about cannabis use and its treatment. As shown in Table 3, GPs rated their knowledge about cannabis *(p* < 0.001), and their skills in screening (*p* < 0.001) and managing cannabis use (*p* < 0.001), higher than nurses rated their own knowledge and skills.

**Table 3:** Self-Assessment of Knowledge about Cannabis Use and Skills Related to Screening and Managing Cannabis Use

**Item**	**Very poor**	**Poor**	**Acceptable**	**Strong**	**Very strong**	***t***	***df***
	***n *(%)**	***n *(%)**	***n *(%)**	***n *(%)**	***n *(%)**		
Knowledge about Cannabis							
GPs (*N* = 501)	35 (7%)	241 (48%)	209 (42%)	14 (3%)	2 (<1%)	3.85**	658
Nurses (*N* = 159)	30 (19%)	76 (48%)	49 (31%)	4 (3%)	0 (0%)		
Skills in Screening for Cannabis Use							
GPs (*N* = 499)	51 (10%)	241 (48%)	187 (37%)	18 (4%)	2 (<1%)	7.32**	654
Nurses (*N* = 157)	57 (36%)	68 (43%)	29 (18%)	3 (2%)	0 (0%)		
Skills in Managing Cannabis Use							
GPs (*N* = 495)	57 (12%)	300 (61%)	127 (26%)	9 (2%)	2 (<1%)		
Nurses (*N* = 156)	55 (35%)	85 (54%)	15 (10%)	1 (<1%)	0 (0%)	7.13**	649

*Note*. *p < 0.01, **p ≤ 0.001.

### Policy attitudes

**Figure 1:**
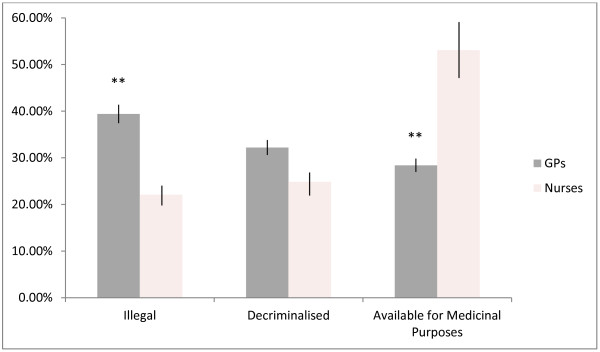
This figure illustrates GP’s and nurses’ percentage agreement with three different cannabis use policy options.

A significantly greater percentage of GPs than nurses reported that cannabis use should be illegal, χ^2^(1) = 14.59, *p* < 0.001, and a significantly greater percentage of nurses than GPs reported that cannabis should be available for medicinal purposes, χ^2^(1) = 30.11, *p* < 0.001, as shown in Figure 1.

### Screening, intervention, and referral

In the previous month, GPs reported screening an average of 3.84 (*SD* = 7.82, *n* = 498) patients for cannabis use and treating an average of 1.71 (*SD* = 4.17, *n* = 500) patients for cannabis use; although, approximately half (46%, *n* = 230) had not engaged in any screening or intervention provision (51%, *n* = 254). Nurses reported screening an average of 2.22 (*SD* = 10.00, *n* = 158) patients for cannabis use and treating an average of 1.33 (*SD* = 8.40, *n* = 159) patients for cannabis use in the last month. Most nurses, however, had not screened (85%, *n* = 134) or provided an intervention to any patients for cannabis use (87%, *n* = 139). In addition, GPs and nurses had referred less than one patient in the last month to an alcohol and other drug (AOD) facility (GPs: *M* = 0.48, *SD* = 1.42, *n* = 500, 374 (75%) had not engaged in referral; Nurses: *M* = 0.84, *SD* = 4.62, *n* = 160, 139 (87%) had not engaged in referral) or to a mental health service (GPs: *M* = 0.76, *SD* = 1.78, *n* = 500, 318 (64%) had not engaged in referral; Nurses: *M* = 0.44, *SD* = 2.39, *n* = 160, 143 (89%) had not engaged in referral). GPs were statistically significantly more likely to have engaged in screening (χ^2^(1) = 72.45, *p* < 0.001), treatment (χ^2^(1) = 64.85, *p* < 0.001), and referral (AOD: χ^2^(1) = 10.21, *p* = 0.001; Mental Health: χ^2^(1) = 38.24, *p* < 0.001) at least once (versus not at all) compared to nurses.

In order to identify if any variables had a unique predictive variance regarding increased provision of screening or intervention for cannabis use, only variables correlated with screening, intervention, and referral to AOD or mental health services at a significance level less than 0.01 were entered into the logistic regression models (Table 4). Interaction terms between health profession and other predictor variables were examined; however, as none were significant they were not included in the reported models. When the significant univariate correlates were included in the regressions, profession remained a significant predictor of screening, intervention and referral to mental health services. GPs were 4.73 times more likely to have engaged in screening provision than nurses, were 4.22 times more likely to have provided an intervention, and were 3.47 times more likely to have referred someone to a mental health service, after controlling for other predictors.

**Table 4:** Binary Logistic Regressions Estimating Screening, Intervention, and Referral Provision in the Previous Month

	**Screening**			**Intervention**			**Referral to AOD Services**			**Referral to Mental Health Services**		
**Item**	**Wald *Χ***^**2**^	***OR***	**99% *CI***	**Wald *Χ***^**2**^	***OR***	**99% *CI***	**Wald *Χ***^**2**^	***OR***	**99% *CI***	**Wald *Χ***^**2**^	***OR***	**99% *CI***
Profession^a^	24.07*	4.73	2.54, 8.79	22.30*	4.22	2.32, 7.67	4.64	1.88	1.06, 3.34	15.52*	3.47	1.87, 6.43
Cannabis Should not be Illegal^b^	--	--	--	--	--	--	--	--	--	5.61	0.57	0.36, 0.91
Cannabis Should not be Decriminalized^b^	0.03	1.04	0.64, 1.70	--	--	--	--	--	--	--	--	--
Cannabis Should not be Available for Medicinal Purposes^b^	7.97*	2.06	1.25, 3.41	1.88	1.34.	0.88, 2.02	--	--	--	0.22	1.13	0.68, 1.86
No Regular User Friend^c^	6.26	0.55	0.35, 0.88	3.22	0.67	0.44, 1.04	7.45*	0.55	0.35, 0.84	7.19*	0.56	0.36, 0.85
Training	2.35	1.33	0.93, 1.90	4.15	1.42	1.01, 1.98	1.25	1.22	0.86, 1.72	0.39	1.11	0.80, 1.55
Perceived Knowledge	0.00	1.00	0.65, 1.53	0.46	1.15	0.77, 1.71	2.37	0.72	0.47, 1.09	0.54	1.16	0.78, 1.75
Perceived Screening Skills	31.79*	3.28	2.17, 4.95	4.28	1.46	1.02, 2.09	8.69*	1.79	1.22, 2.65	4.54	1.49	1.03, 2.16
Perceived Management Skills	1.82	1.38	0.87, 2.20	3.13	1.47	0.96, 2.26	0.70	1.21	0.78, 1.88	1.14	1.27	0.82, 1.95
10 Minute Brief Assessment Can Reduce Use	1.09	1.11	0.91, 1.36	2.70	1.17	0.97, 1.42	--	--	--	--	--	--
Effective Psychological Treatments Exist	1.03	1.12	0.90, 1.38	0.17	1.04	0.85, 1.28	2.06	1.17	0.94, 1.45	--	--	--
People in My Position Are Effective	0.46	0.93	0.76, 1.14	0.65	1.08	0.89, 1.31	--	--	--	0.42	1.06	0.88, 1.28

*Note.* -- indicates a variable not included in a model. Superscripts represent the reference group for each independent variable: ^a^ GPs, ^b^ Should be… (illegal, decriminalized, or available for medicinal purposes), ^c^ Has a friend who is a regular cannabis user. * p < 0.01.

Confidence in the provision of screening was important, with every unit increase in perceived screening skills associated with a 3.28 times greater chance of having engaged in screening provision in the previous month and an 8.69 times greater chance of having referred a patient to an AOD service. Neither the degree of training, knowledge, nor perceived skills in cannabis intervention, however, offered a significant unique prediction of screening, intervention, or referral.

Health professionals who had a friend who regularly used cannabis were 1.80 to 1.83 times more likely to have referred someone to an AOD or mental health service than those without such a friend. In regards to attitudes, participants who believed cannabis should not be available for medicinal purposes were 2.06 times more likely to screen for cannabis use than those who believed it should be available. While that attitude was a significant univariate correlate of intervention and referral to a mental health service, it did not provide a unique prediction of those actions.

### Barriers and facilitators

As shown in Table 5, GPs more often than nurses reported not having enough time for screening and intervention provision (*p* < 0.001), not being personally interested in screening and treatment provision (*p* = 0.001), not believing that cannabis use is the most important clinical issue (*p* < 0.001), not wanting to attract more cannabis users to their clinic (*p* < 0.001), and believing that the effort required to motivate a cannabis user to quit is not justified by the likelihood of a positive outcome (*p* < 0.002). Nurses more often reported that cannabis users should only be treated by specialists in the field (*p* < 0.001). In respect to facilitators, GPs more so than nurses reported that having more referral options would facilitate their provision of screening and intervention for cannabis use (*p* < 0.001).

**Table 5:** **Barriers and Facilitators for Cannabis Use Screening and Intervention (*N*** **= 664)**

**Statement**	**GPs agreement *n *(%)**	**Nurses agreement *n *(%)**	**χ**^**2**^**(1)**
Barriers			
Do not have the skills to screen or provide an intervention	336 (67%)	124 (77%)	5.94
Cannabis use often is not the most important issue that needs to be addressed during a patient’s visit	286 (57%)	56 (35%)	23.80**
Patients may not be receptive to screening or intervention	279 (55%)	98 (61%)	1.45
Do not have time to screen or provide an intervention	242 (48%)	41 (25%)	25.56**
Do not have support from colleagues/organization to screen or provide an intervention	138 (27%)	55 (34%)	2.68
The effort required to motivate a cannabis user to quit is not justified by the likelihood of a positive outcome	101 (20%)	15 (9%)	9.80*
Do not have a personal interest in providing screening or treatment for cannabis use	95 (19%)	12 (7%)	11.78**
Do not have professional interest in providing screening or treatment for cannabis use	77 (15%)	13 (8%)	5.45
I do not want to attract more cannabis users to my clinic	76 (15%)	5 (3%)	16.41**
Most people who use cannabis do not need screening or treatment	30 (6%)	7 (4%)	0.61
Cannabis users are unpleasant to work with	24 (5%)	3 (2%)	2.64
Cannabis users should only be treated by specialists in the field	15 (3%)	18 (11%)	17.36**
Facilitators			
More training	408 (81%)	144 (89%)	6.03
Access to up-to-date management guidelines and recommendations	396 (79%)	130 (81%)	0.30
Having more options for referrals	391 (78%)	88 (55%)	32.31**
Having more resources to assist me (e.g., web interventions)	315 (63%)	103 (81%)	0.10
Believing that screening and intervention would lead to positive outcomes	232 (46%)	67 (42%)	1.00
Believing that screening and intervention are clinically important	134 (27%)	51 (32%)	1.54
Nothing would facilitate the screening or intervention of cannabis use	6 (1%)	2 (1%)	<0.01

*Note*. *p < 0.01, **p ≤ 0.001.

## Discussion

This study evaluated GP’s and nurses’ perceived knowledge, beliefs, and behaviors toward cannabis use and its screening and management. As a whole, the findings suggest that despite GPs and nurses having little to no training in cannabis-related issues, most are aware of basic treatment-related issues and believe that it is part of their role to be educated about cannabis use. Yet, many GPs and nurses believed their knowledge about cannabis use and their skills in screening and managing cannabis use to be poor. The findings also suggest that nurses may be less knowledgeable about cannabis use and feel less role legitimacy for its screening and treatment. Thus, it is not surprising that being a GP was consistently associated with cannabis service provision compared with being a nurse. When controlling for other relevant variables, GPs were three to five times more likely than nurses to have engaged in screening, brief intervention, and referral to mental health services. Study findings also indicate that policy attitudes, personal experience, and perceived screening skills may influence service provision.

The finding that nurses perceive themselves to be less knowledgeable and to have less role legitimacy than GPs is consistent with alcohol use research [16,25]. While GPs rated their knowledge and skills better than did nurses, it is important to note that less than half of GPs considered their knowledge and screening skills to be acceptable to very strong and less than one-third considered their skills in managing cannabis use to be acceptable to very strong. In addition, only a quarter of GPs and nurses reported an accurate estimate of the chance of developing dependence [6,7]. As 21% of participants did not answer the question, 24% may overestimate the actual rate of accurate responses, as those who did not know the accurate figure may have chosen to skip this item. Both GPs and nurses also overestimated the effectiveness of pharmacological treatments [31]. These findings indicate that even though nurses may benefit from training in cannabis-related issues more so than GPs, training also has the potential to improve GP’s service provision to cannabis users.

Consistent with previous research [18,20-22], GPs and nurses identified not having enough skills, patients not being receptive, and not having enough time as common reasons for not engaging in screening and intervention for cannabis use. Not believing that cannabis use is the most important issue was a new common barrier identified in this study. Given that more GPs than nurses endorsed this barrier, it is not surprising that GPs were more likely than nurses to report not having enough time and that having more referral options would facilitate their service provision. These findings suggest that GPs may see more legitimacy for their role in screening than they do intervention provision. As this study did not examine health practitioners’ beliefs about their role legitimacy for screening, future research would benefit from examining role legitimacy beliefs for both screening and intervention provision.

Prior to this study, scant research had statistically examined whether personal views and experience with cannabis use influenced service provision. An exception is Johnson and colleagues’ study [18], which found that primary care physicians who had family and friends with substance use problems had less difficulty discussing substance use with patients. The current study found that having a friend who regularly uses cannabis increased referral provision, but not screening and intervention provision. This study also identified that compared to health practitioners who believed that cannabis should be available for medicinal purposes, those who believed cannabis should not be available for medicinal purposes were twice as likely to have screened a patient for cannabis use. Along with Lock and colleagues’ [21] finding that nurses’ beliefs about alcohol’s perceived benefits affect their willingness to provide alcohol-related services, these findings suggest that training in cannabis-related issues should incorporate discussions on the effectiveness of cannabis for medicinal purposes and how personal experience can shape their professional behavior. Doing so may help to motivate all health practitioners to engage in evidenced-based service provision.

### Implications for policymakers and educators

General practitioners and nurses represent the overwhelming majority of primary care providers in Australia. They have enormous potential to reach a broad range of people for early intervention, where the focus is on prevention and health promotion. Although Australian GPs are being encouraged via government reimbursement schemes to provide brief advice in many prevention areas, including drug and alcohol use [13], efforts to increase service provision for cannabis users may need to be directed at both nurses and GPs, in order to utilize the opportunity for collaboration between GPs and nurses in the primary care setting. There are several reasons why nurses should be involved in this process. First, nurses were consistently less likely to have engaged in service provision than GPs. Second, GPs in this study were substantially more likely to identify barriers to service provision that are difficult to resolve, such as not having enough time and cannabis use not being the most important issue. Third, in 2010 the Australian Government announced a $523 million investment in Australia’s nurses in order reduce GP’s clinical burden [32]. Such an investment increases the need to improve nurses’ skills to ensure that they are fully able to undertake effective preventive health activities. Although the aim of the initiative is to allow GPs to focus on diagnosis, while nurses focus on tasks such as care co-ordination, health assessments, and health education [32], the most suitable model of practice for nurses has not been established. Thus, when developing training models, researchers will need to keep in mind that nurses may be operating from a substitution model (only doing those tasks delegated to them) or from a collaborative model (working autonomously within a practice). In addition, researchers will need to find an effective method for resolving the low legitimacy that nurses feel for managing cannabis use.

Hence, part of a training strategy may require education and empowerment of nurses in terms of their clinical potential to assist in managing cannabis use problems. In a substitution model, it may be advantageous to educate both GPs and nurses in how best to utilize their respective clinical strengths. This might involve nurses performing the screening and assessment of patients’ readiness for change and then referring motivated patients on to GPs for a brief intervention. GPs may develop clinical plans that involve utilizing nurses in their role as health educators. This would allow GPs to remain in the referral process, but minimize their clinical burden, while appropriately managing patients concerns. In those circumstances where nurses and nurse practitioners are working in a relatively autonomous context, nurses could be trained in both screening and intervention, referring to the GP only when the extremity of clinical features might warrant it.

Regardless of whom efforts are focused on, combining motivational interviewing with screening and intervention training for cannabis use may lead to greater provision of services to individuals who use cannabis. Research has found that motivating GPs to provide an intervention does not need to involve long professional development sessions. For example, a 20-minute motivational discussion with GPs has substantially increased their likelihood of discussing methods for managing dependent cannabis users [30]. Such motivational training may circumvent the negative effect that health practitioners’ pre-existing low motivation has on service provision. Providing GPs with training and support has been found to only increase provision of alcohol screening and brief advice for those GPs who were already motivated to work with patients with alcohol problems prior to receiving training [28]. Almost all GPs and nurses in the current study reported it was part of their role to receive education about cannabis use, and over three-fourths reported that receiving more training would increased their service provision. As such, providing training in screening (that includes information about screening resources, practice guidelines, and referral options) and in brief motivational interventions (that includes information about treatment resources, and discussion of personal experience and medicinal cannabis use) during professional development seminars may increase service provision for cannabis users.

When evaluating the effects of training on GPs’ and nurses’ service provision, researchers should carefully monitor increases in knowledge and attitudes toward service provision. Previous research found that training in a brief intervention led to increases in the understanding of a brief intervention for nurses, but not GPs [33]. Furthermore, while nurses’ knowledge about alcohol increased as a result of training, their positive attitudes towards discussing alcohol with patients decreased. Thus, training needs to be carefully developed and administered to avoid potential negative consequences.

### Strengths and limitations

The strength of the study is its large sample size. Limitations include the sole recruitment of health practitioners from *Healthed* seminars, reliance on self-reports of service provision, using a survey without established psychometric properties, and the low response rate (35%). Notably, the obtained survey response rate was similar to other surveys conducted with Australian GPs [34,35], and thus, may not be indicative of non-respondents being less interested in cannabis use than responders. An additional limitation of this study is the high amount of missing data regarding the percentage of patients who will develop dependence (21% missing data). Research suggests that the high nonresponse on this item may have been due to the cognitively demanding nature of the question [36]. In order to reduce the non-response rate for this item, future researchers may want to adapt the open-ended item to include response options. Lastly, respondents were not provided with operational definitions for screening, brief interventions, or referrals; therefore, these items may have been interpreted differently. In light of these limitations, results may only generalize to GPs and nurses interested in women’s and children’s health who also have an interest in contributing to research. Future research with professionals interested in men’s health and that uses operational definitions and a validated survey may obtain different results.

## Conclusions

This study found that as a group, GPs and nurses perceive their knowledge and skills pertaining to cannabis-related issues to be poor. In addition, this study identified that health professionals’ occupation, attitudes, personal experience, and skills may affect their provision of cannabis-related care. Education and training, therefore, may be particularly important to improving service provision for individuals who use cannabis, especially training that utilizes the complementary roles that each professional plays in general practice, with a special emphasis on increasing nurses’ knowledge, skills, and role legitimacy.

## Abbreviation

GPs: General Practitioners.

## Competing interests

Dr Ramesh Manocha is the CEO of *Healthed* pty ltd, a company that conducts educational seminars for GPs, nurses and other health professionals. This study was conducted at *Healthed* seminars. The other authors do not have any conflicts of interest.

## Authors’ contributions

MMN, PG, PD, and RM contributed to the development of the study survey. MN wrote the first draft of the article and conducted all statistical analyses. PG revised the first draft and provided statistical advice. PD, RM, DJK, and JC contributed to other manuscript revisions. All authors read and approved the final manuscript.

## Authors’ information

MMN is a clinical psychologist and senior lecturer. PG is a doctoral student in Community Medicine and Public Health. PD is a drug and alcohol educator. DJK is a clinical psychologist and professor. RM is a general practitioner and senior lecturer. JC is a professor who has a PhD in Community Medicine and Public Health.

This work was carried out at the National Cannabis Prevention and Information Centre at the University of New South Wales. All surveys were distributed during *HealthEd* seminars conducted in major cities across Australia.
